# Intracellular and extracellular domains of protein tyrosine phosphatase PTPRZ-B differentially regulate glioma cell growth and motility

**DOI:** 10.18632/oncotarget.2366

**Published:** 2014-08-19

**Authors:** Annika M. Bourgonje, Anna C. Navis, Jan T.G. Schepens, Kiek Verrijp, Liesbeth Hovestad, Riet Hilhorst, Sheila Harroch, Pieter Wesseling, William P.J. Leenders, Wiljan J.A.J. Hendriks

**Affiliations:** ^1^ Department of Cell Biology, Radboud University Medical Center, Nijmegen, The Netherlands; ^2^ Department of Pathology, Radboud Institute for Molecular Life Sciences, Radboud University Medical Center, Nijmegen, The Netherlands; ^3^ PamGene International BV, 's-Hertogenbosch, The Netherlands; ^4^ Department of Neuroscience, Institut Pasteur, Paris, France; ^5^ Department of Pathology, VU University Medical Center, Amsterdam, The Netherlands

**Keywords:** PTPRZ1, diffuse infiltrative growth, signal transduction, cell migration, tyrosine phosphorylation, PDZ

## Abstract

Gliomas are primary brain tumors for which surgical resection and radiotherapy is difficult because of the diffuse infiltrative growth of the tumor into the brain parenchyma. For development of alternative, drug-based, therapies more insight in the molecular processes that steer this typical growth and morphodynamic behavior of glioma cells is needed. Protein tyrosine phosphatase PTPRZ-B is a transmembrane signaling molecule that is found to be strongly up-regulated in glioma specimens. We assessed the contribution of PTPRZ-B protein domains to tumor cell growth and migration, via lentiviral knock-down and over-expression using clinically relevant glioma xenografts and their derived cell models. PTPRZ-B knock-down resulted in reduced migration and proliferation of glioma cells *in vitro* and also inhibited tumor growth *in vivo*. Interestingly, expression of only the PTPRZ-B extracellular segment was sufficient to rescue the *in vitro* migratory phenotype that resulted from PTPRZ-B knock-down. In contrast, PTPRZ-B knock-down effects on proliferation could be reverted only after re-expression of PTPRZ-B variants that contained its C-terminal PDZ binding domain. Thus, distinct domains of PTPRZ-B are differentially required for migration and proliferation of glioma cells, respectively. PTPRZ-B signaling pathways therefore represent attractive therapeutic entry points to combat these tumors.

## INTRODUCTION

Diffuse gliomas comprise a heterogeneous group of glial brain tumors that share high migratory potential, as evidenced by diffuse infiltration in the surrounding brain parenchyma along white matter tracts and blood vessels [1]. This phenotype severely reduces efficacy of conventional therapeutic strategies, i.e. surgery and radiotherapy [2]. A hallmark of the highly malignant WHO-grade-IV gliomas, with a very dismal prognosis of less than 15 months [3], is the additional local occurrence of angiogenesis. This angiogenesis has been combated using specific inhibitors in phase III trials, but thus far therapeutic survival benefit is lacking because the approach leaves the diffuse infiltrative component unaffected or even increases diffuse growth [4-6]. Additional treatment modalities aiming at diffuse tumor cells that escape surgery and radio-chemotherapy, hence knowledge about the molecular mechanisms that underpin glioma cell migration and proliferation in the brain parenchyma, are therefore urgently needed.

Aberrant phosphotyrosine-based signaling is a hallmark of cancer, and gliomas are no exception; tyrosine kinase membrane receptors like EGFR, ERBB2, PDGFRA, MET and VEGFR2 have been implicated in glioma growth, angiogenesis and cell motility [7]. A role for the counter-acting protein tyrosine phosphatase (PTP) enzyme family [8, 9] is much less studied. There is compelling evidence that PTPs influence cell migration [10], especially during neuronal development [8, 9]. Several PTPs have been linked to carcinoma development [11, 12] and their involvement in glioma biology is gradually being uncovered [13]. PTEN, for example, is inactivated or absent in one-third of high-grade gliomas, resulting in increased PI3K-mediated cell proliferation and survival [7, 14]. For multiple additional PTPs the contribution to glioma etiology, especially the diffuse infiltration in the neuropil, requires further study.

Within the receptor-type PTP (RPTP) subfamily there is a number of transmembrane enzymes that resemble cell adhesion molecules, having extracellular domains that might engage in interactions with neighboring cells or extracellular matrix components [8-10]. These characteristics make RPTP genes, including *PTPRZ1*, potentially important regulators of cell motility and growth. *PTPRZ1* encodes three isoforms (PTPRZ-A, PTPRZ-B and phosphacan) that share a carbonic anhydrase-like (CAH) and a fibronectin type III (FNIII) domain at the protein's N-terminus [15]. Furthermore, a spacer with chondroitin sulfate proteoglycan attachment sites is present in isoforms PTPRZ-A and phosphacan. PTPRZ-B lacks most of this spacer, resulting in a smaller extracellular part. PTPRZ-A and PTPRZ-B have identical intracellular parts consisting of a catalytically active membrane-proximal and an inactive membrane-distal PTP domain. The phosphacan isoform lacks these PTP domains and represents a secreted protein [15]. Several PTPRZ-interacting proteins have been identified. For instance, the extracellular ligand pleotrophin binds to and inactivates PTPRZ, thereby increasing the phosphorylation of intracellular substrates β-catenin [16], Fyn [17], β-adducin [18] and Alk [19]. Additional interaction partners include contactin-1, which binds to the CAH domain [20], and tenascin-C and -R that bind to the FNIII domain [21]. It is thought that these proteins form complexes with the extracellular matrix [22] to induce and facilitate migration.

PTPRZ expression, in particular PTPRZ-B [23], is up-regulated in glioma tumor specimens [24-26]. *PTPRZ1* knock-down in glioblastoma cell lines reduced cell migration [25] and tumor growth [27], and PTPRZ overexpression enhanced cell migration [24]. However, these cell models produce circumscribed tumors that lack the highly invasive phenotype when grown orthotopically [28]. Furthermore, PTPRZ protein domains that steer glioma cell behavior still need to be uncovered. Here we investigated the role of PTPRZ and its protein domains, exploiting glioma models that faithfully recapitulate diffuse infiltrative growth *in vivo* [28-30]. Lentivirus-mediated knock-down and subsequent rescue experiments revealed that PTPRZ-mediated effects on migration rely exclusively on its extracellular domain, whereas impact on proliferation depends on the intracellular carboxyl-terminal PDZ domain binding site. These findings identify PTPRZ as a dual entry point for glioma therapy development.

## RESULTS

### Modulation of PTPRZ-B expression levels in glioblastoma cells

**Figure 1:**
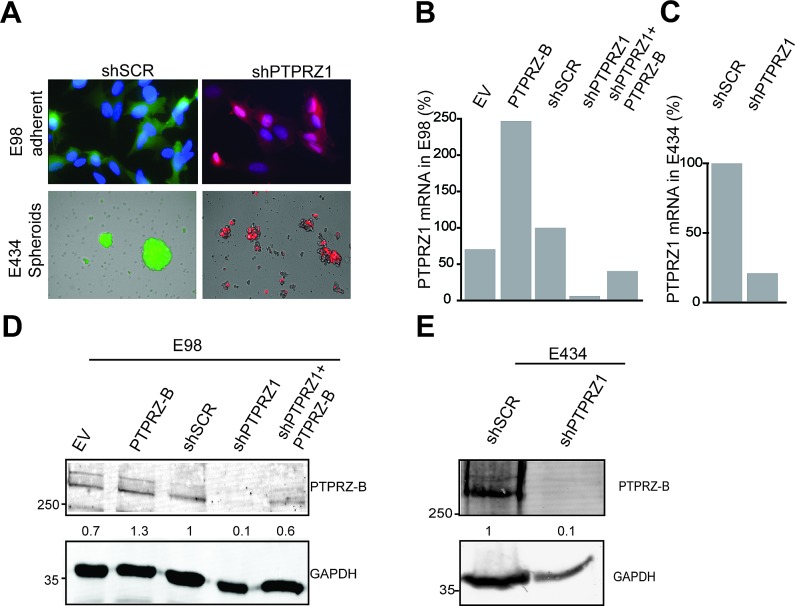
*PTPRZ1* expression or knock-down in E98 and E434 cells A) Fluorescent images of glioma cells containing shSCR or shPTPRZ1 knock-down constructs carrying GFP or TagRFP fluorescent reporters, respectively. E98 cells were DAPI counterstained. B) *PTPRZ1* mRNA levels in lentivirally transduced E98 cells were determined by qRT-PCR. Expression, normalized to β-actin, is given as percentage of that in control shRNA-expressing (shSCR) cells. EV; empty vector control. C) Normalized *PTPRZ1* mRNA levels in lentivirally transduced E434 spheroids, determined by qRT-PCR. D) E98 cells were lentivirally transduced with expression constructs for PTPRZ-B protein or EV and/or *PTPRZ1* or control (SCR) shRNAs. After three days, lysates were analyzed on immunoblots using PTPRZ-B (upper) and GAPDH (lower) antibodies. Normalized PTPRZ-B levels, relative to that in shSCR control lysates, are depicted in between the blot images. Molecular weight indications (in kDa) are on the left. E) Immunoblot analysis (as in D) of E434 lysates 72 hrs after lentiviral transduction with the indicated constructs.

In line with previous reports [24-26], high *PTPRZ1* expression levels are detectable in glioma tumors (data not shown) and in human xenograft-derived cells in culture (Fig. 1). The two well-characterized glioma xenograft lines E98 and E434 [28] differ in their *in vitro* culture regimen; anaplastic oligodendroglioma-derived E434 cells only propagate under neurosphere growth conditions, using serum-free neurobasal medium [31], whereas glioblastoma-derived E98 cells additionally grow in standard DMEM/10%FCS as an adherent monolayer (Fig 1A). To assess PTPRZ influence on glioma growth and migration, lentiviral vectors for PTPRZ-B expression and *PTPRZ1* shRNA-mediated knock-down (targeting all three isoforms) were generated (supplementary Fig. S1). We introduced a silent mutation in the PTPRZ-B open reading frame to create an shRNA-insensitive lentiviral PTPRZ-B expression construct and used this throughout for validation and rescue purposes. Following lentiviral transduction of E98 and E434 cells with *PTPRZ1* shRNA, a five- to twenty-fold reduction of *PTPRZ1* transcript levels (Fig. 1B,C) and a five- to ten-fold drop in PTPRZ-B protein content (Fig. 1D,E) was obtained. As for C6 glioma cells [23], it is the short transmembrane variant PTPRZ-B that was detected in E98 and E434 lysates (Fig. 1D,E). Use of the lentiviral PTPRZ-B expression vector resulted in PTPRZ-B protein levels that were one to three times that of the endogenous protein, also in presence of *PTPRZ1* shRNA (Fig. 1D).

### PTPRZ-B stimulates glioma cell growth *in vitro*

**Figure 2:**
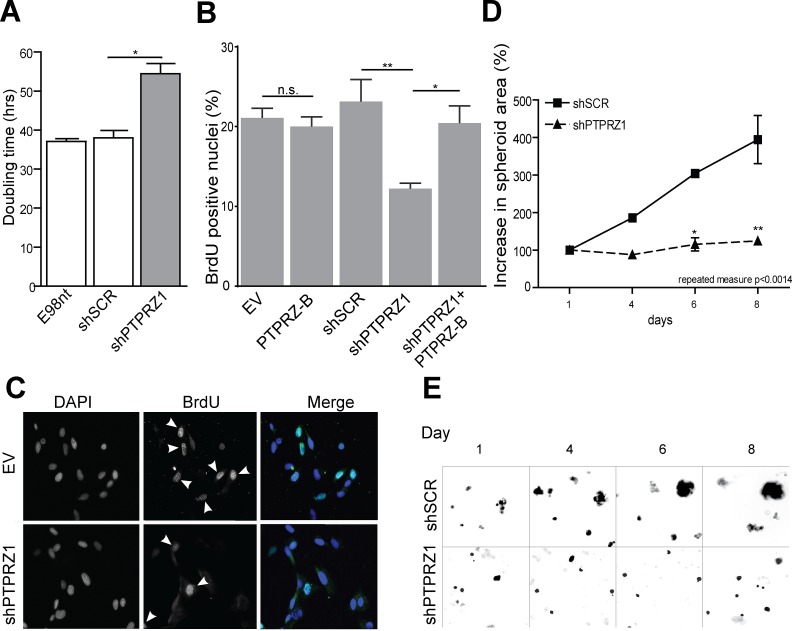
*PTPRZ1* knock-down impairs glioma cell growth A) E98 cells expressing shSCR or shPTPRZ1 knock-down constructs and non-transduced (E98nt) controls were cultured in microtiter plates, total cell mass was determined at various time points, and population doubling times were calculated from resulting curves. Error bars indicate SD, asterisk indicates p<0.05 (Student *t*-test, n=3). B) Transduced E98 cells were grown on coverslips and labeled with BrdU for 1 hr. Percentage of BrdU-positive nuclei among DAPI-stained cells was determined (n>3). Error bars indicate SD. Asterisks reflect confidence levels (ANOVA; * p<0.05; ** p<0.01; n.s., not significant). C) Representative images from (B), showing DAPI and BrdU (arrowheads) positivity. D) Transduced E434 cells were seeded into a 96-well plate and images were collected at indicated time points. Increase in spheroid size relative to day 1 is depicted. Error bars indicate SEM and asterisks indicate significance levels (* p<0.05; ** p<0.01). Repeated measure ANOVA: p<0.0014. E) Representative images from (C), exploiting TagRFP and GFP autofluorescence in shRNA vectors.

PTPRZ-B over-expression and knock-down effects on E98 and E434 cell proliferation was assessed via direct measurement of cell content and via BrdU incorporation. Sulforhodamine B (SRB) proliferation assays revealed that *PTPRZ1* knock-down significantly inhibited growth of adherent E98 cells, as illustrated by an increased cell doubling time (Fig. 2A). Accordingly, BrdU pulse-labeling showed a significant reduction of the percentage of S-phase cells in shPTPRZ1-transduced samples (p<0.05). PTPRZ-B over-expression did not significantly affect E98 proliferation, in agreement with the modest increase in PTPRZ-B levels on immunoblot. Nevertheless, *PTPRZ1* knock-down effects were fully rescued by PTPRZ-B re-expression (Fig. 2B).

To investigate whether observed *PTPRZ1* knock-down effects are on the level of cell viability, apoptosis or cell cycle progression, BrdU pulse-labeled cells were immunostained for BrdU, for the G1-S-G2-M marker Ki-67 and for cleaved Caspase-3. The percentage of apoptotic cells was less than 1% for all conditions (data not shown) and, with the exception of *PTPRZ1* knock-down cells, approximately 20% of cells ended up BrdU-positive. Intriguingly, always half of the cells stained positive for Ki-67, even among shPTPRZ1-transduced cells of which 10% is in S-phase (Fig. 2B-C). This suggests that the growth impairment upon *PTPRZ1* knock-down reflects increased duration of cell cycle time.

Knock-down of *PTPRZ1* also resulted in significantly delayed growth in E434 cells. Since these cells can only be propagated as spheroids, we measured proliferation capacity by means of the spheroid diameter (Fig 2D-E). Fluorescent protein signals in the lentivirally transduced cells were used to image spheroid size over time. Whereas shSCR-transduced E434 spheroids showed clear signs of growth, shPTPRZ1-transduced spheroids only increased a little in size.

### PTPRZ-B facilitates migration of E98 and E434 glioma cells

**Figure 3:**
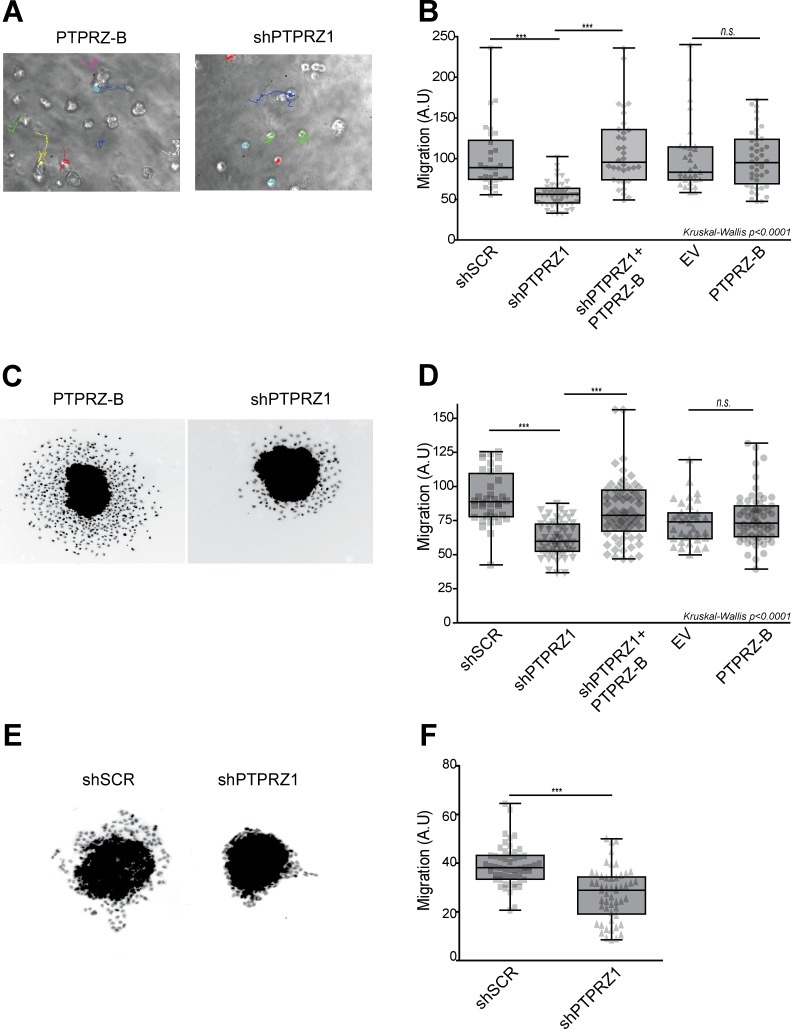
*PTPRZ1* knock-down reduces glioma cell migration A-B) E98 cells were lentivirally transduced with indicated protein (PTPRZ-B), EV control or shRNA (shPTPRZ1 or shSCR control) expression vectors, and seeded in an agarose/Matrigel 3D matrix. A) Migration paths were recorded overnight and analyzed by single-cell tracking. B) Averaged migration speeds (arbitrary units, AU) of individual cells, normalized to shSCR (n>30), are shown (gray points). Box-plot whiskers represent minimum and maximum. Asterisks indicate significance (*** p<0.001; n.s., not significant; Kruskal-Wallis p<0.0001). C) E98 spheroids were seeded on a Matrigel layer and fixed and DAPI-stained 24hrs later. Two representative images are shown. D) Pixel distance from the spheroid border was calculated for individual cells and per spheroid (n>32) average outgrowth was plotted (grey data points). Box-plot whiskers represent minimum and maximum. Asterisks indicate significance (*** p<0.001; n.s., not significant; non-parametric ANOVA: Kruskal-Wallis p<0.0001). E) Representative pictures of transduced E434 spheroids, fixed and DAPI-stained, after 24hrs on Matrigel. F) Mean pixel distance that cells traveled from the spheroid border was calculated, and averages per spheroid (n>53) are plotted (grey data points). Box-plot whiskers represent minimum and maximum, and asterisks indicate significance (***p<0.001).

We next investigated whether *PTPRZ1* influence on cell migration [24, 25] is represented in our glioma models. Following *PTPRZ1* knock-down, migration of individual E98 cells in a 3D Matrigel/agarose environment was significantly reduced (p<0.001), an effect that was rescued by expression of the shRNA-insensitive PTPRZ-B transcript in knock-down cells (Fig. 3A-B). To confirm this in a different setting, we generated transduced E98 spheroids using the hanging-drop method. Resulting homogeneously sized E98 spheroids were subsequently placed on a thin Matrigel layer for 24 hours and migratory performance of outgrowing cells, expressed as distance travelled from the edge of the spheroid [32], was calculated semi-automatically. Again, *PTPRZ1* knock-down cells migrated significantly less (p<0.05) than scrambled controls, and restoration of PTPRZ-B levels rescued this phenotype (Fig. 3C-D). Of note, PTPRZ-B over-expression by itself did not alter E98 motility. In a similar fashion, outgrowth of lentivirally transduced E434 spheroids was inhibited by PTPRZ knock-down (p<0.05; Figure 3E-F). Interestingly, whereas outgrowing E98 cells presented as scattered, amoeboid-like migrating cells, the migrating E434 cells largely remained interconnected, suggestive of collective migration [33]. Taken together, the reduced migration *in vitro* due to *PTPRZ1* knock-down, which was rescued following PTPRZ-B re-expression, corroborates a facilitating role for PTPRZ-B in cell motility.

### PTPRZ1 knock-down impairs glioma cell growth in mouse brain

**Figure 4:**
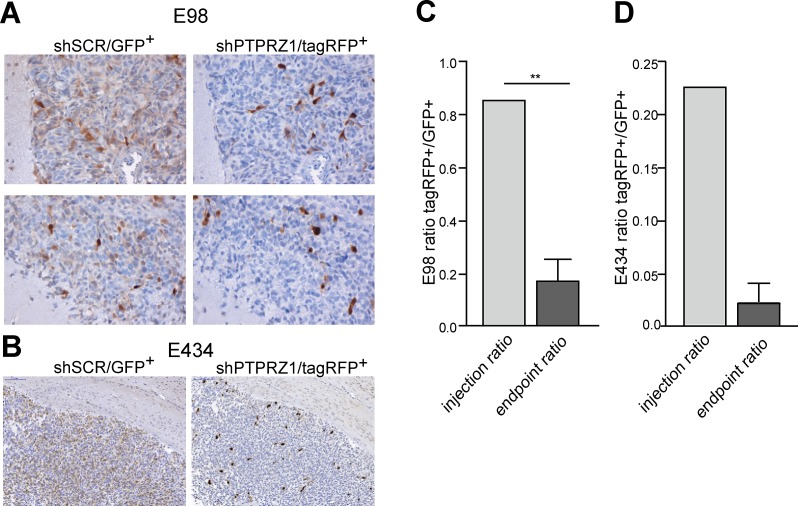
*PTPRZ1* knock-down impairs glioma growth *in vivo* Suspensions of E98 or E434 cells expressing *PTPRZ1* shRNA and TagRFP (shPTPRZ1/TagRFP^+^) or control shRNA and EGFP (shSCR/EGFP^+^) were mixed and injected intracerebrally in immunodeficient mice. TagRFP^+^/EGFP^+^ ratios were calculated using fluorescent images. A) For E98 this resulted 3-4 weeks later in compact (top panel) and diffuse infiltrative (bottom panel) tumor areas. Representative areas, immunohistochemically stained for TagRFP or EGFP and haematoxylin-counterstained, of one out of three animals are shown. B) E434 glioma tumor-bearing mice were processed 9 weeks post-injection. Representative pictures of one out of two animals are shown. For E434 a 50% transduction efficiency after two consecutive transduction rounds was achieved resulting in also non-transduced cells being injected. C) Quantification of TagRFP^+^/EGFP^+^ ratios in E98 xenografts, as determined for five different tumor areas per animal, averaged and compared to the ratio pre-injection. Error bar represents SD. One-sample Student's *t*-test yielded p<0.01 (**). D) Quantification of TagRFP^+^/EGFP^+^ ratios in E434 xenografts and pre-injection mixture. Error bar indicates SD.

To investigate how these PTPRZ-B knock-down effects translate to *in vivo* tumor behavior, we co-injected intracerebrally shPTPRZ1- and shSCR-expressing E98 cells that were tagged with TagRFP and EGFP fluorescent marker proteins, respectively. When cultured under serum conditions, E98 cells grow to compact non-diffuse tumors upon orthotopic injection, but when cultured as spheroids they display diffuse infiltrative properties (our unpublished results). Stably transduced E98 cells were therefore grown as spheroids in serum-free neurobasal medium for at least two weeks prior to injection. E434 cells exclusively grow as spheroids in neurobasal medium and were directly injected after dissociation. ShPTPRZ1/TagRFP and shSCR/EGFP spheroids were processed to single cell suspensions and a 1:1 mixture of both cell types was injected intracerebrally in immunodeficient mice. Animals were sacrificed when symptoms of tumor burden appeared, and brains were examined for the distribution of EGFP- and TagRFP-containing cells (Fig. 4). EGFP-positive control cells greatly outnumbered the TagRFP-expressing *PTPRZ1* knock-down cells in the tumors (p<0.01), in accordance with the low *in vitro* proliferation rate of the PTPRZ1 knock-down. This *in vivo* proliferation impairment due to *PTPRZ1* knock-down likely obscured migratory effects; the amount of TagRFP-positive (knock-down) cells relative to haematoxilin-stained nuclei in E98 tumors was similar in compact and diffuse infiltrative areas (0.11 ± 0.03 SEM versus 0.09 ± 0.01 SEM, respectively; p<0.61). Also for EGFP-positive (control) cells the relative contribution to compact and diffuse infiltrative tumor areas was comparable (0.38 ± 0.04 SEM versus 0.39 ± 0.08 SEM, respectively; p<0.93). Collectively, *in vitro* and *in vivo* data demonstrate a significant reduction of tumor growth upon *PTPRZ1* knock-down, prompting us to address the molecular mechanisms by which PTPRZ-B impacts on glioma cell behavior.

### The PTPRZ-B PDZ binding domain mediates growth stimulatory signals

**Figure 5:**
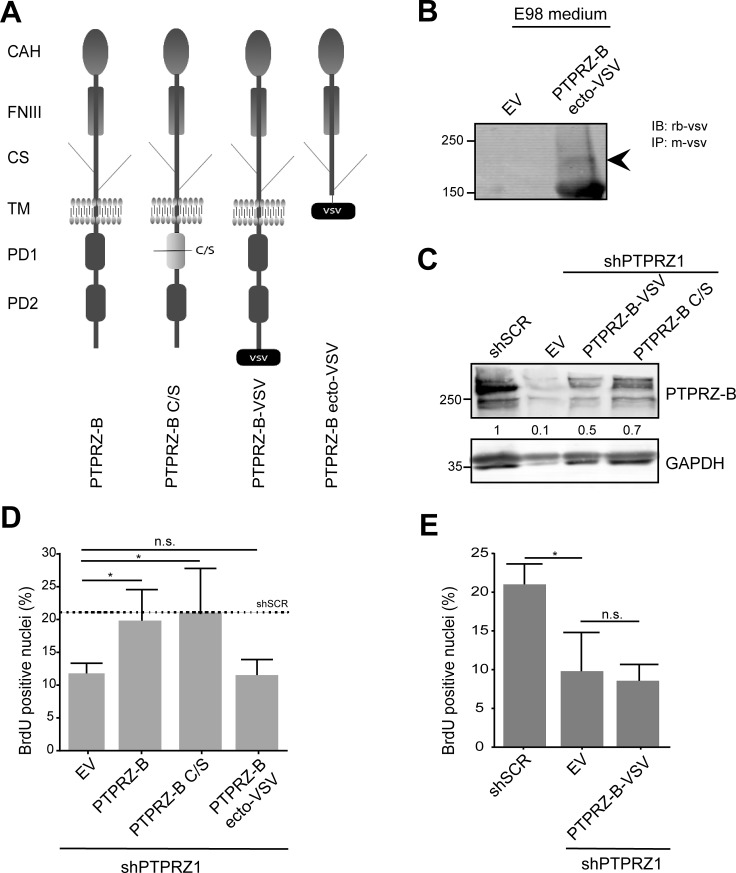
The PTPRZ-B C-terminus is required to rescue the proliferation phenotype in E98 *PTPRZ1* knock-down cells A) Schematic representation of PTPRZ-B variants used in the study. CAH, carbonic anhydrase-like; FNIII, fibronectin type-III; CS, chondroitin sulfate chain; TM, transmembrane region; PD1, phosphatase domain 1; PD2, inactive phosphatase domain 2; VSV, C-terminal VSV-G epitope tag. C/S indicates the mutation of the catalytic site cysteine to serine that renders PD1 inactive. B) Conditioned medium from E98 cells expressing PTPRZ-B ecto-VSV or from empty vector (EV) control cells was subjected to immunoprecipitation using anti-VSV monoclonal antibody and subsequent immunoblot analysis using rabbit antiserum against VSV. Molecular weight markers (in kDa) are indicated on the left. Arrowhead on the right indicates the chondroitin sulfated PTPRZ-B extracellular part. C) E98 shPTPRZ1 cells were transduced with the indicated expression constructs and 72 hrs later lysates were prepared and analyzed on western blots using PTPRZ-B antiserum. GAPDH staining served as loading control and E98 shSCR lysate was included for comparison. Normalized PTPRZ-B levels, relative to that in shSCR control lysates, are depicted in between the blot images. D) E98 shPTPRZ1 cells were seeded on collagen-coated glass coverslips and hours later, transduced with the indicated expression constructs. Three days after transduction cells were labeled with BrdU, fixed and stained. Fluorescent images were taken and the percentage of BrdU-positive nuclei among DAPI-stained cells was determined (n>3). Error bars indicate SD. Confidence levels, as determined by ANOVA, are represented by asterisks (* p<0.05; n.s., not significant). The dashed line indicates the percentage in E98 shSCR cells as obtained in comparable experiments. E) Percentage of BrdU-positive nuclei in DAPI-stained E98 or E98 shPTPRZ1 cells following transduction with the indicated constructs (n=3). Error bars indicate SD. Confidence levels, as determined by ANOVA, are represented by an asterisk (* p<0.05; n.s., not significant).

PTPRZ-B may exert its stimulatory effect on E98 glioma proliferation in multiple ways, potentially involving extracellular CAH, FNIII and chondroitin sulfate-containing domains or intracellularly the two PTP domains and the C-terminal PDZ domain target site (Fig. 5A). Only the first, membrane-proximal PTP domain in PTPRZ-B is enzymatically active [34]. Because the PTPRZ-B knock-down phenotype in E98-shPTPRZ1 cells could be rescued with an shRNA-insensitive full-length expression construct (Figs. 2-3), we tested the contribution of the individual PTPRZ-B protein domains using this assay. Three different shRNA-resistant PTPRZ-B cDNA versions were generated (Fig. 5A). In PTPRZ-B ecto-VSV the transmembrane and intracellular PTPRZ-B segments were replaced by a VSV-G epitope tag, effectively leading to the secretion of a C-terminally tagged PTPRZ-B extracellular domain (Fig. 5B). PTPRZ-B C/S represents a cysteine-to-serine catalytically inactive mutant, and in PTPRZ-B-VSV the ‘SLV_COOH_’ PDZ domain target site is blocked by a C-terminal VSV-G tag. Expression of all constructs was readily detected in E98-shPTPRZ1 cells (Fig 5B-C).

BrdU pulse-labeling experiments revealed that reduction in percentage of cells in S-phase, due to knock-down of endogenous PTPRZ, was fully rescued by full-length PTPRZ-B and the inactive PTPRZ-B C/S mutant. Expression of PTPRZ-B ecto-VSV or PTPRZ-B-VSV did not rescue the knock-down phenotype (Fig. 5D-E). Thus, PTPRZ-B impacts on cell cycle progression through mechanisms that are independent of PTPRZ-B phosphatase activity and require interactions with the PTPRZ PDZ domain target site.

### Extracellular PTPRZ-B interactions impact on glioma cell migration

**Figure 6:**
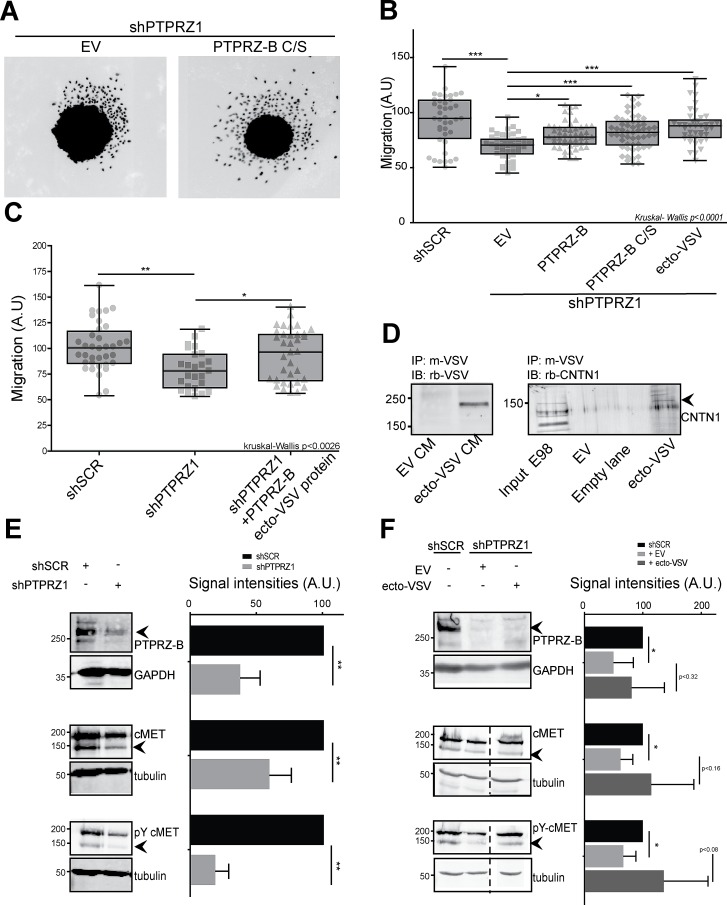
The PTPRZ-B ectodomain stimulates glioma migration A) E98 or E98 shPTPRZ1 cells were lentivirally transduced with indicated constructs. Three days later spheroids were generated and the next day seeded on a thin Matrigel layer. After being cultured for another 24 hrs cells were fixed and stained with DAPI. Pictures were collected and two representative images are shown. B) Using these images the migration, in arbitrary units, was calculated per spheroid (light grey data points) as the average distance cells traveled from the spheroid border using FIJI-based software. Box-plot whiskers represent minimum and maximum values. Significance levels are indicated by asterisks (* p<0.05; ** p<0.01; *** p<0.001; Kruskal-Wallis p<0.0001). C) Using the above set-up, spheroid migration was also determined following addition of immunopurified PTPRZ-B ecto-VSV to E98 shPTPRZ1 cells (shPTPRZ1 +PTPRZ-B ecto-VSV protein) during the 24 hrs of culturing on Matrigel. Kruskal-Wallis p<0.003. D) PTPRZ-B ecto-VSV was immunopurified, using mouse anti-VSV monoclonal antibody-coupled beads, from conditioned medium (CM) of HEK-293FT cells transfected with PTPRZ-B ecto-VSV (ecto-VSV) or empty vector (EV) expression plasmids (left panel). Subsequently, beads were incubated with E98 whole cell lysates and co-purifying proteins were analyzed on western blots using Contactin-1 antiserum (right panel). E) Lysates of E98 cells, that were transduced with indicated shRNA constructs and blasticidin-selected, were prepared and analyzed on immunoblots. Molecular weight markers (kDa) are indicated on the left. Upper panels: PTPRZ-B immunostaining, using GAPDH as loading control. Middle and lower panels: blots were probed with antisera against MET (middle) and Y1234-1235-phosphorylated MET (lower) while tubulin staining served as control. Representative images are shown. On the right, the quantification (n>3) of normalized signal intensities (arbitrary units) is given. Error bars indicate SD and asterisks reflect significance based on one-sample Student *t*-test (** p<0.01). F) E98 shPTPRZ1 cells were transduced with indicated constructs and 72 hrs later whole cell lysates were prepared and analyzed as described under (E). E98 shSCR cell lysate was included for comparison. Representative images are shown on the left and quantification (n>3) of normalized signal intensities (arbitrary units) is given on the right. Dashed lines indicate removal of in-between lanes from the depicted blots. Error bars indicate SD and asterisks reflect significance (* p<0.05, Student *t*-test).

Rescuing abilities of PTPRZ-B variants were also assessed in the spheroid migration assay. Migratory impairment due to *PTPRZ1* knock-down in E98-shPTPRZ1 cells was effectively rescued upon re-expression of full-length PTPRZ-B, PTPRZ-B C/S and, importantly, also by PTPRZ-B ecto-VSV (Fig. 6A-B). Moreover, purified VSV-tagged PTPRZ-B ectodomains, isolated from conditioned medium of transfected HEK-293FT cells, also rescued the PTPRZ knock-down effect on E98 cell migration (Fig. 6C). Contactin-1 has been put forward as a membrane-anchored PTPRZ ligand, binding to the CAH domain and impacting on cell migration [22]. In line with this, endogenous contactin-1 from E98 cells indeed bound to PTPRZ-B ecto-VSV that was preloaded onto anti-VSV antibody-coupled beads (Fig. 6D), underscoring PTPRZ-B – contactin-1 interplay in E98 glioma cells.

Our results did not disclose PTPRZ phosphatase activity as a regulator of glioma cell migration and proliferation. It is reasonable to assume, however, that the phosphotyrosine-controlled activity of cellular kinases is affected following *PTPRZ1* knockdown in E98 cells. A comparison of E98-SCR and E98-shPTPRZ1 cell extracts in a tyrosine kinase microarray activity assay [35] revealed that *PTPRZ1* knock-down cells displayed reduced activity, most notably towards PDGFRB- and MET-derived peptides (Supplementary Fig. S2). We previously reported on the importance of MET for E98 cell migration [32]. Immunoblot analyses confirmed that in shPTPRZ1-expressing E98 cell lysates MET tyrosine phosphorylation was reduced four-fold (Fig. 6E). Additionally, *PTPRZ1* knock-down also resulted in lower MET protein levels, suggesting that PTPRZ-B regulates MET transcription, synthesis or degradation. Interestingly, *PTPRZ1* knock-down effects on MET are partly alleviated by PTPRZ-B ecto-VSV expression (Fig. 6F). Taken together, results demonstrate a dual oncogenic role for *PTPRZ1* in glioma cells. The PTPRZ-B intracellular PDZ domain binding site is required to orchestrate a submembranous complex that boosts cell proliferation, while the extracellular portion binds receptors and adhesion molecules on nearby cells and stimulates migration.

## DISCUSSION

Effective treatment of glioma patients requires that also tumor cells that diffusely spread out into the brain parenchyma are therapeutically addressed. In the current study we assessed the contributions of different PTPRZ-B domains to glioma cell migration and proliferation using models that faithfully recapitulate infiltrative tumor growth. We show that *PTPRZ1* knock-down in E98 glioblastoma and E434 oligodendroglioma cells results in impaired growth and motility *in vitro* and reduced tumor growth *in vivo*, also in diffuse infiltrative tumor areas. Furthermore, our rescue experiments disclose a dyad functionality for this RPTP. The intracellular PTPRZ-B C-terminal PDZ domain binding site, and not its enzymatic PTP activity, turned out essential for effects on cell proliferation. Conversely, the PTPRZ-B extracellular moiety, which in part resembles the naturally occurring phosphacan isoform, impacted on cell migration.

*PTPRZ1* is upregulated in gliomas, which likely contributes to enhanced tumor cell migration [24-26]. A role for PTPRZ in proliferation was less clear. Reduction of tumor growth as well as absence of growth effects have been reported for *PTPRZ1* knock-down experiments [25, 27]. Administration of soluble PTPRZ ectodomain is known to inhibit proliferation of oligodendroglial precursor cells [36]. In our study, the PTPRZ-B ectodomain did not affect the reduction of E98 cells in S-phase that resulted from *PTPRZ1* knock-down. Addition of wild-type or enzymatically inactive PTPRZ-B did suffice to rescue proliferation impairment, but a C-terminally tagged version did not. If PTPRZ phosphatase activity would have been crucial for glioma cell proliferation, expression of an inactive ‘substrate protection’ mutant in *PTPRZ1* knock-down cells might act dominant-negative and worsen the effect. On the contrary, proliferation rescue by the catalytically dead PTPRZ-B mutant and not by the C-terminally tagged version rather implicates PTPRZ-B's C-terminal protein interaction potential as important for signaling complexes that steer glioma proliferation. As yet we cannot rule out that also protein interactions mediated by PTPRZ-B domains other than its C-terminal PDZ domain binding motif contribute to its proliferation signaling mode.

Multiple PDZ-containing proteins have been reported to bind to the PTPRZ C-terminus; PSD95 [37], MAGI-3 [38], MAGI-1, GOPC, Mupp1, Synj2bp, Snta1, Sntb1 and Veli-3 [39]. This opens up several mechanisms by which PTPRZ may influence proliferation. For instance, tyrosine kinase ErbB4 auto-activation is suppressed by PSD95 through PDZ domain-mediated interactions with both the enzyme PTPRZ and the substrate ErbB4 [40]. Likewise, PTPRZ and its substrate β-catenin form a complex through MAGI-1 [39, 41]. However, current findings argue against an enzymatic role for PTPRZ in such PDZ-based complexes and rather point to a scaffolding role. By clustering proteins submembranously in glioma cells, PTPRZ might contribute to efficient funneling of growth stimulatory signals towards the cell's interior.

In both our glioblastoma and anaplastic oligodendroglioma model, the PTPRZ ectodomain did not impinge on proliferation but rather was instrumental for migration. Also in U87-MG glioblastoma cells, PTPRZ positively influenced adherence and migration [24] and an antibody targeting the PTPRZ extracellular region delayed U87-MG compact tumor formation *in vivo* [42]. PTPRZ ectodomain binding partners include extracellular matrix components like pleiotrophin, tenascin-C and tenascin-R, and cell surface molecules [36] like contactin-1 [20, 21]. Extracellular binding of PTPRZ and contactin-1 is hypothesized to recruit additional proteins, such as tenascins, to the complex [22]. In oligodendroglial precursors this inhibits proliferation and triggers differentiation towards myelinating cells. In our glioma models, PTPRZ-contactin-1 interactions apparently lead to migratory responses. This difference may result from separate PTPRZ-mediated interactions in either system or, alternatively, by absence or presence of facilitator proteins involved. Contactin-1, tenascin-C and tenascin-R have adhesion and migration effects in glioma cells [21, 24, 25, 42, 43].

Recently we showed that MET inhibition by cabozantinib effectively stops E98 cell migration [32]. Here, migration impairment in E98-shPTPRZ1 cells co-incided with reduced MET activity. For a candidate substrate, rather increased phosphorylation levels are expected upon knock-down of PTPRZ, and thus far MET does not meet PTPRZ substrate criteria [44]. Together with ErbB and PDGFR family members, MET represents one of the oncogenic drivers in glioma tumor biology [7]. Whereas E98 cells displayed a scattered, single-cell migration pattern in spheroid outgrowth experiments, E434 cells showed a more collective migratory behavior. This may reflect MET signaling differences in the two models, bearing in mind ‘scatter factor receptor’ as alternative name for MET. In our *in vivo* experiments we were unable to assess PTPRZ-B's migratory role on tumor formation because this was blurred by effects on cell proliferation. Current findings now provide a basis to experimentally separate both type of effects through independent targeting of PTPRZ-B intracellular and extracellular binding potential. Further knowledge on PTPRZ intracellular and extracellular signaling involvement will strengthen its candidacy as a therapeutic target in gliomas.

## MATERIALS AND METHODS

Experimental details on cell lines and antibodies used, plasmid construction, lentiviral transduction, immuno-blotting, -precipitation and –histochemical procedures, peptide microarray analysis, and statistical analyses are provided as supplementary material.

### Cell proliferation and viability assays

E98 cells were grown on collagen I-coated (10 μg/cm^2^; Invitrogen) coverslips to 60-80% confluency, and incubated for 1hr with culture medium containing BrdU (50 μM; Life Technologies, #B23151). Cells were then washed three times with PBS and fixated in 2% paraformaldehyde (PFA) in 0.1 M phosphate buffer (PB: 46 mM NaH_2_ PO_4_; 354 mM Na_2_HPO_4_, pH 7.4). Subsequently, coverslips were washed with PBS and quenched with 50 mM NH_4_Cl in PBS. Cells were blocked and permeabilized for 1hr in blocking buffer (5% Normal Goat Serum and 0.1% Triton X-100 in PBS) and then incubated for 2.5hrs with anti-BrdU, anti-Ki67 or anti-cleaved Caspase 3 antibodies in blocking buffer with added DNAseI (100 ng/mL; Roche) and MgCl_2_ (2.5 mM) at 37 °C. Bound antibodies were detected with goat-anti-mouse Alexa 488 and goat-anti-rabbit Alexa 647 secondary antibodies in PBS. Coverslips were mounted on microscope slides in DAPI-containing Mowiol (Sigma-Aldrich) and images were collected on a Leica DMRA Fluorescence microscope, equipped with a DFC340 FX CCD camera, using 40x and 63x objectives. DAPI- and BrdU-positive nuclei and cleaved Caspase 3-positive cells were counted using FIJI software [45].

For proliferation assays, stably transduced E98 cells were seeded in triplicate in a 96-well microtiter plate (10,000 cells/well), and cultured in 10% FCS-containing DMEM. At various time points, cells were washed with PBS and fixed using 10% trichloroacetic acid for 1hr at 4 °C, washed with water and afterwards frozen until all time points were collected. Cells were then stained with 1% Sulforhodamine B (SRB; Sigma-Aldrich) in 1% acetic acid for 20 minutes. Plates were subsequently washed with 1% acetic acid and dried at 60 °C for 3 hrs. Protein-bound SRB was then dissolved in 10 mM Tris (pH 10.5) and absorption was measured at 510 nm using a micro-plate reader (Bio-Rad).

Lentivirally transduced E434 spheroids were seeded in a 96-well imaging plate (BD Biosciences, #353219) and allowed to settle overnight. The next day, images were taken using a BD Pathway 855 high-content bio-imager (BD Biosciences). Fluorescent images (TagRFP or EGFP-based) were taken longitudinally at days 4, 6 and 8 and the average increase in spheroid area was calculated automatically using FIJI software. After images had been taken on the fourth day, half of the medium was replenished and spheroid growth was continued.

### 2D and 3D migration assays

E98 spheroids were generated by the hanging-drop method. In brief, 0.7 mL methylcellulose (12 mg/mL; Sigma, M6385) was added to 4.3 mL E98 cell suspension in DMEM supplemented with 10% FCS (500,000 cells total) and 25 μL drops were seeded in a dry culture dish. The dish was then inverted and incubated overnight at 37 °C in the presence of 5% CO_2_. The next day, individual spheroids were seeded in a 96-well imaging culture dish (BD Falcon, #353219) coated with Matrigel (30 μg/mL PBS; BD Biosciences, #356237) and further incubated in DMEM with 10% FCS, at 37 °C and 5% CO_2._ For E434, cultured spheroids were directly plated onto Matrigel-coated wells containing B27-supplemented neurobasal medium. After 24 hrs, wells were washed with PBS and cells were fixed with 2% PFA in 0.1 M PB, followed by DAPI staining. Fluorescent images were collected on a high-content microscope (Leica DMI6000B) extended with a motorized x-y scanning stage (Leica EL6000 illumination source), and FIJI software [45] was used to automatically quantify spheroid outgrowth. Briefly, cells which migrated from the spheroids were selected, coordinates of each individual cell were determined, and distance from the spheroid border was calculated using the coordinates and radius of the spheroid. For each experiment the average cell migration distance across all spheroids (n>31) was calculated.

Cell cultures in solidified Matrigel/Agarose matrices were prepared as follows. 10 μL Matrigel and 5 μL 2% agarose (Seaplaque Agarose; Lonza #50101 in PBS) were mixed with 35 μL 10% FCS-containing DMEM and 50 μL of Matrigel/agarose mix was added to the wells of a 96-well microtiter plate which was placed on ice. One minute later, 50 μL cell suspension (50,000 cells) was mixed resulting in a 3D environment containing cells. Cells were imaged overnight with 10-min intervals on a time lapse system (Nikon Diaphot 300 with Hamamatsu C8484-05G CCD Camera, Okolab 4 well CO_2_ stage incubator and Okolab 2D time lapse software) at 37 °C and 5% CO_2_ with a 10x objective. Cells were manually tracked for at least 2 hrs using the FIJI plug-in [45].

### Intracerebral injection of spheroid-derived cells

All animal experiments were approved by the local Animal Experimental Committee of the Radboud University Medical Center. Athymic female BALB/c nu/nu mice (18–25 gram, age 6–8 weeks) were kept under specified pathogen-free conditions and received food and water *ad libitum*. E98 and E434 cells were grown as spheroids in supplemented neurobasal medium for at least two weeks prior to orthotopic injection [29]. A 20 μL cell suspension (10^7^ cells/mL in PBS) containing a mixture of shSCR/GFP and shPTPRZ1/TagRFP labeled cells was injected per animal (n=5 and 4 for E98 and E434 cells, respectively). Prior to injection, the ratio of GFP- and TagRFP-positive cells was determined using an EVOS fluorescence imaging system (AMG) and FIJI Software. Animals were closely monitored and sacrificed when signs of tumor burden (especially weight loss and neurological dysfunction) were observed. Brains were harvested, and parts were either formalin-fixed and paraffin-embedded (FFPE) or snap-frozen in liquid nitrogen, and stored for (immuno)histochemical analysis.

## SUPPLEMENTARY MATERIAL, FIGURES AND TABLES


