# Supplementary material for: Intracellular and extracellular domains of protein tyrosine phosphatase PTPRZ-B differentially regulate glioma cell growth and motility
Source: Oncotarget. 2014 Aug 19;5(18):8690–702. doi: 10.18632/oncotarget.2366 (PMC4226714; doi:10.18632/oncotarget.2366)
Supplement: Supplementary file 1 [file oncotarget-05-8690-s001.pdf]

Intracellular and extracellular domains of protein tyrosine phosphatase PTPRZ-B differentially regulate glioma cell growth and motility

Supplementary Material

Cell lines and antibodies

Generation and maintenance of orthotopic glioma xenograft models E98 (glioblastoma) and E434 (anaplastic Oligodendroglioma) have been described previously [1]. E98 and E434 orthotopic xenograft-derived cells were cultured as spheroids in neurobasal medium supplemented with B27 Supplement, 2 mM L-glutamin, 2 μg/mL Heparin, 1% penicillin/streptomycin (all Gibco), 20 ng/mL EGF and basic-FGF (both PromoCell) [2]. Regular passaging of spheroids was done by gentle mechanical dissociation. In addition, E98 cells were grown as adherent cultures in DMEM supplemented with 10% FCS, and passaged using trypsinisation [3]. E434 cells cannot be propagated under these conditions.

Antibodies used were as follows: for Western Blotting: mouse anti-PTPRZ (1:1000; BD Biosciences, #610179), mouse anti-VSV (IP: 5μL; P5D4 [4]), mouse anti-tubulin (1:5000; DSHB, University of Iowa, E7), rabbit anti-MET (1:2000; Cell Signaling Technology, #8198), rabbit anti-pMET (1:2000; Cell Signaling Technology, #3027), rabbit anti-GAPDH (1:5000; Cell Signaling Technology #2118), rabbit anti-Contactin-1 (1:2000) [5], for immunohistochemistry mouse anti-BrdU (1:50; Sigma-ALDRICH, #2531), rabbit- ki67 (1:200 Clone SP6; Thermo Scientific #Rm-9106) rabbit-cleaved caspase 3 (1:200; Cell Signaling Technology, #9661) rabbit anti-GFP (1:1000) [6], and rabbit anti-TagRFP (1:500; Evrogen, AB233). As secondary antibodies, IRDye 680- or 800-conjugated goat anti-mouse and goat anti-rabbit antibodies (1:20,000; LI-COR Bioscience), Alexa Fluor 488 goat-anti-mouse and Alexa Fluor 647 goat-anti-rabbit antibodies (1:300; Alexa) or biotinylated goat-anti-rabbit IgGs (1:200; Dako) were used.

Plasmid constructs

The plasmid pLenti6/BLOCK-iT-DEST (Invitrogen) was adapted to also contain CMV promoter-driven TagRFP or EGFP expression cassettes. Briefly, the CMV-TagRFP cassette was PCR-amplified from pTagRFP-C (Evrogen) using oligonucleotide set 1 (all oligonucleotide sequences are listed in Supplementary Table 1), digested with *Xba*I and *Xho*I, and ligated into *Xba*I/*Xho*I-digested pLenti6/BLOCK-iT-DEST. Likewise, the CMV-driven EGFP expression cassette was amplified from pEGFP-N2 (Clontech) using oligonucleotide

set 2, digested with *Xba*I and ligated in the *Xba*I-linearized pLenti6/BLOCK-iT-DEST. For over-expression purposes, the mouse phosphoglycerate kinase (PGK) promoter was inserted upstream of the *att*R1 recombination site in these pLenti6/ BLOCK-it-DEST-TagRFP and - EGFP variants. Briefly, oligonucleotide set 3 was used to amplify the pLenti6.2/V5-DEST (Invitrogen) PGK fragment and *the Cla*I digested amplicon was ligated to produce plasmids pLenti6/PGK-DEST-TagRFP and pLenti6/PGK-DEST-EGFP. To create knock-down constructs, shPTPRZ1 (set 4) and shSCR (set 5) oligonucleotide heteroduplexes were ligated in pENTR/U6 vector and subsequently Gateway-cloned into pLenti6/ BLOCK-iT-DEST- TagRFP (for shPTPRZ1) and pLenti6/ BLOCK-iT-DEST-EGFP (for shSCR) according to the manufacturer's protocols (Invitrogen).

A pENTR/U6 derivative was customized by inserting oligonucleotide heteroduplex set 6 between the unique *Sal*I-*Xba*I sites, rendering pENTR/*Not*I-*Xho*I. To clone PTPRZ-B cDNA (Refseq NM_001206838.1), we first tailored pENTR/*Not*I-*Xho*I by inserting an *Sst*II site-containing linker (set 7) in the unique *Hind*III site. The PTPRZ-B open reading frame was produced by reverse-transcriptase PCR using oligonucleotide set 8 and E98 total RNA as template. The *Sst*II and *Not*I digested cDNA was subsequently inserted into the *Sst*II site-containing pENTR/*Not*I-*Xho*I variant, resulting in plasmid pENTR-wtPTPRZ-B. A shPTPRZ1-resistant PTPRZ-B version (further indicated as pENTR-PTPRZ-B) was created via introduction of a silent C-T mutation at nucleotide position 1861 (numbering according to NM_001206838) in the shPTPRZ1 recognition site, using oligonucleotide set 9 and the Quickchange site-directed mutagenesis kit (Stratagene) according to manufacturer's instructions.

PTPRZ-B cDNA was subsequently adjusted via site-directed mutagenesis to encode an enzymatically inactive PTPRZ-B C/S mutant (GC to CG, at positions 3613-3614) using oligonucleotide set 10 and the afore-mentioned protocol. Furthermore, a C-terminally VSV-tagged full-length PTPRZ-B variant was generated by first generating a *KpnI* site (AGTTTAA to GGTA, pos. 4757-4763) using oligonucleotide set 11, and subsequently introducing oligonucleotide heteroduplex set 12, encoding an in-frame C-terminal VSV-G epitope tag (flanked by *Kpn*I sites). To enable expression of the PTPRZ-B ecto-domain only, first a *Kpn*I site was created at the codon preceding the PTPRZ-B transmembrane-encoding region (AGTTATA to GGTA, pos. 2726-2732) using oligonucleotide set 13 in the mutagenesis protocol. Subsequent *Cla*I digestion and re-ligation resulted in removal of residues 776-1448 comprising the PTPRZ-B intracellular domains. Finally, the new *Kpn*I site was used to insert the VSV-G epitope tag-encoding heteroduplex 12. All resulting pENTR-

PTPRZ-B plasmid variants were sequence-verified before being used in Gateway LR cloning reactions with pLenti6/PGK-DEST-TagRFP as destination vector. Also using Gateway cloning, the empty pENTR/*Not*I-*Xho*I vector served to generate pLenti6/PGK-EV-TagRFP as empty vector control.

Lentiviral transduction of glioblastoma cells and spheroids

Lentiviruses were produced using HEK-293FT cells according to the manufacturer's instructions (Invitrogen). Briefly, 95% confluent 10cm culture dishes with HEK-293FT cells were transfected overnight, using JetPRIME reagent (Westburg) and the appropriate plasmid cocktail. The next day, medium was refreshed and 48-72 hrs later virus-containing medium was harvested, passed through a 0.45 µm pore size filter and stored at -80 °C. E98 Glioma cells or E434 spheroids were transduced by adding virus-containing medium to the cultures, at a 1:2 to 1:5 virus to medium ratio. After an overnight incubation, cells were superinfected with virus to increase the percentage of transduced cells. Routinely, this led to 80-100% transduction efficiency for E98 cells and 40-80% for the spheroid E434 cultures. Stably transduced E98 cells were selected by adding Blasticidin (2 µg/mL; Invitrogen). For rescue experiments, cells were first transduced twice with shPTPRZ1-expressing lentiviruses. Several days later, two or three rounds of transduction with PGK promoter-driven rescue constructs were performed and cells were subjected to the proliferation and migration assays 72 hrs later.

Real-time quantitative RT-PCR

Total RNA was isolated using RNA-Bee (Tel-Test Inc. cs104B) using standard trizol-chloroform extraction methods, and concentrations were measured spectrophotometrically. Reverse transcriptase reactions were performed using Iscript^{tm} cDNA synthesis kit (Bio-Rad) according to the supplier's specifications. Specificity and efficacy of real-time quantitative PCR primer pairs for *PTPRZ1* and β-*actin* (Qiagen) have been verified previously by Schmidt *et al.* [7]. Reactions, containing 3 µL of cDNA, 1 µL of the pre-mixed primer pair, 5 µL of SYBR Green PCR master mix (Bio-Rad) and 1 µL MQ, were run on a CFX96^{tm} Real Time system using the C1000^{tm} Thermal Cycler (Bio-rad). Reactions were initialized at 95 °C for 15 minutes and then cycled 40 times at 95 °C for 15 s and 60 °C for 40 s. After the last cycle, a dissociation curve was recorded between 60 °C and 95 °C with and increment of 0.5 °C.

The amount of *PTPRZ1* RNA was determined relative to *ACTB* levels using the delta Ct method [8].

Immunoblotting and immunoprecipitation

Cells were washed with cold PBS and scraped in ice-cold lysis buffer (50 mM Tris-HCl, pH 7.5; 150 mM NaCl; 1% Triton X-100 (Serva); 1 mM PMSF; 100 mM NaF; 2 mM Na_3VO_4; 20 mM $Na_4P_2O_7$; complete protease inhibitor cocktail, Roche, #13006200 (1 tablet in 1.5 mL MQ, 20 μL solution per mL lysis buffer)). Samples were either further processed for immunoprecipitation (see below) or added to 2x SDS sample buffer (60 mM Tris-HCl, pH 6.8; 2% SDS; 100 mM dithiothreitol; 0.001% bromophenol blue; 10% glycerol) and heated at 95 °C for 5 min. Protein samples were size-separated using SDS-PAGE on 8% gels and electro-blotted onto PVDF membrane (Immobilon-FL, #IPFL00010) according to standard protocols. Membranes were blocked using 1% BSA in TBST (10 mM Tris-HCl, pH 8.0; 150 mM NaCl; 0.1% Tween-20 (Sigma-Aldrich)) for 1 hr at RT, and subsequently incubated with primary antibodies in 1% BSA in TBST, overnight at 4 °C. Membranes were then washed three times for 10 min in TBST and subsequently incubated with the appropriate secondary antibodies for 1 hr at RT in the dark. Blots were analyzed using the Odyssey imaging system (LI-COR Biosciences). Immunoblot images were analyzed quantitatively using standard FIJI software.

To visualize PTPRZ-B ecto-VSV in E98 conditioned medium, immunoprecipitation was performed. 30 μL ProtG sepharose beads (GE Healthcare, #17-0618-01) were incubated with mouse-VSV antibody overnight at 4 °C. Beads were washed 5 times with TBS and incubated with E98 conditioned medium overnight at 4 °C. Beads were washed 5 times with TBS and suspended in SDS sample buffer. After centrifugation, the supernatant was used for gel loading and blotted as described above. For purification purposes PTPRZ-B ecto-VSV was produced in HEK-293FT cells transfected with pLenti6/PGK-PTPRZ-B ecto-VSV-TagRFP, using JetPRIME according to the manufacturer instructions. The secreted PTPRZ-B ectodomain was purified from conditioned medium via immunoprecipitation using mouse anti-VSV bound to ProtG sepharose beads, as described above. VSV-tagged proteins were eluted from the beads using excess VSV peptide [9].

For co-immunoprecipitation, mouse anti-VSV was coupled to ProtG sepharose beads by overnight rotation at 4 °C in TBS. After 5 subsequent washes with TBS, conditioned medium from HEK293FT cells transfected with either pLenti6/PGK-EV-TagRFP or pLenti6/PGK-PTPRZ-B ecto-VSV-TagRFP was added to the beads allowing coupling to

VSV overnight at 4 °C. After 5 washes, E98 cell lysates (prepared as described above) were added to the beads and were incubated overnight at 4 °C. The next day, beads were washed 5 times with TBS before being taken up in 2x SDS sample buffer. Samples were processed for immunoblotting as described above.

Immunohistochemistry

FFPE sections of 4 μm were subjected to immunohistochemical stainings according to standard procedures [10]. In brief, sections were de-paraffinized in xylene and rehydrated in PBS. Endogenous peroxidases were blocked in 3% H_2O_2 in PBS, followed by epitope retrieval (10 min boiling in 10 mM sodium citrate, pH 6.0). Slides were then washed twice in PBS, blocked in 20% normal serum (from the species in which the secondary antibody was raised) in PBA (1% BSA in PBS) for 20 min, and incubated overnight at 4 °C with primary antibodies in PBA. After 3 PBS washes, sections were incubated with biotinylated secondary antibodies in PBA for 1hr at room temperature. Slides were washed 3x with PBS and signals were then enhanced by incubation with Avidin/Biotin complexes (Vector Laboratories, #PK-6100), both 1:100 in PBA for 45 min, followed by 3 PBS washes and visualization of specific signals using 3,3′-diaminobenzidine (Bright-DAB, ImmunoLogic, #B04). All sections were counterstained with haematoxylin and mounted in Quick-D Mounting medium (Klinipath BV, #7280).

Quantification of immunodetected EGFP and TagRFP signals was done using KS400 software (Carl Zeiss AG) and a custom-written macro. Sections of FFPE brains with orthotopic glioma xenografts were included in the analysis (n=3 and 2 for E98 and E434, respectively) and at least five non-overlapping microscopic fields (magnification x200) were measured per immunostaining for each animal. The TagRFP- or GFP-positive area per tumor field was divided by the total tumor area as determined via nuclear haematoxylin staining, and average values per animal were determined and used to calculate TagRFP/GFP ratios. Ratios were compared to those prior to injection using the one-sample Student's *t*-test.

Peptide microarray analysis

E98 cells stably expressing shSCR/GFP and shPTPRZ1/TagRFP constructs were grown to 80% confluency in 6-well plates (4 wells per sample). Cells were washed twice with ice-cold PBS prior to lysis with M-PER Mammalian Extraction Reagent supplemented with protease and phosphatase inhibitor cocktails (Thermo Scientific) for 30 min at 4 °C. Lysates

were centrifuged (15 min, 14,000 rpm, 4 °C), and supernatants were snap frozen in liquid nitrogen and stored at -80 °C. Protein concentrations were determined using the BCA protein assay (Thermo Scientific). Kinase activity measurements were performed in quadruplicate on Tyrosine kinase PamChip arrays on a PamStation 12 instrument (PamGene International BV) essentially as described [11]. Sample input was 5 µg per array. A Student's *t*-Test was used to identify the peptides that are significantly ($p<0.05$) different between the treatments.

Statictical analysis:

Statistical analysis was performed using GraphPad Prism 5 or PamGene's proprietary BioNavigator software. For two conditions the Student *t*-test was applied. For other conditions ANOVA, repeated measure ANOVA or non-parametric Kruskal-Wallis with post-hoc tests were performed. When all experimental conditions were included, Tukey's multiple comparison test was used. In contrast, when comparisons were made to a single "control" experimental condition the Dunn's post-hoc test was applied.

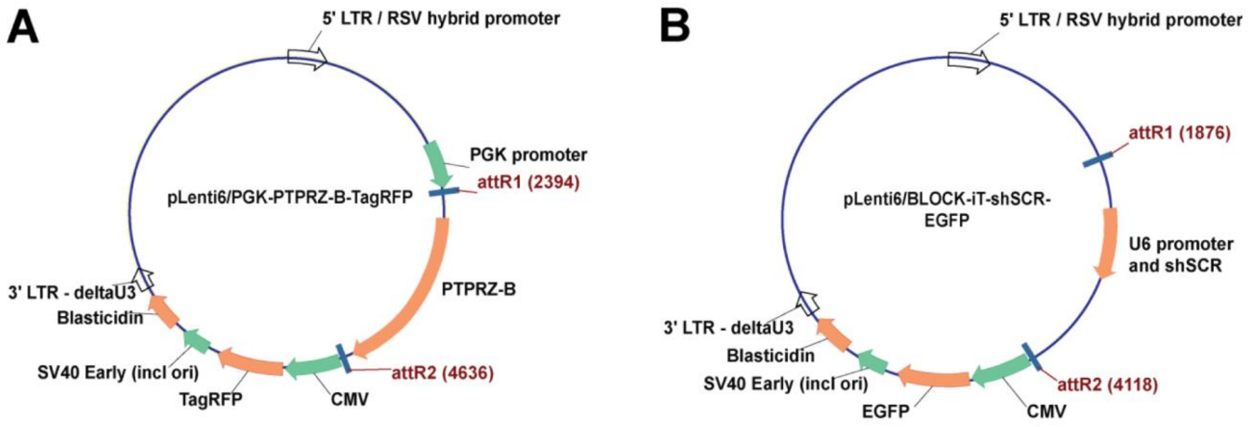

Supplementary Figure S1: Plasmid maps of constructs used to create lentiviral vectors for glioma cell transduction. A) Schematic overview of the expression construct that is generated in an LR Gateway cloning reaction, using pLenti6/PGK-DEST-tagRFP and the appropriate cDNA-containing pENTR plasmid (in this case full-length PTPRZ-B). B) Schematic overview of the scrambled control knock-down construct that resulted from an LR Gateway reaction involving the pLenti6/BLOCK-iT-DEST variant (with added fluorescent protein expression cassette) and the pENTR/U6 plasmid with inserted oligonucleotide heteroduplex for shSCR production. Long terminal repeat (LTR) regions, attR1 and attR2 Gateway recombination sites, and the CMV promoter-driven TagRFP and EGFP expression cassettes as well as the SV40 early promoter-driven Blasticidin selection cassette are indicated.

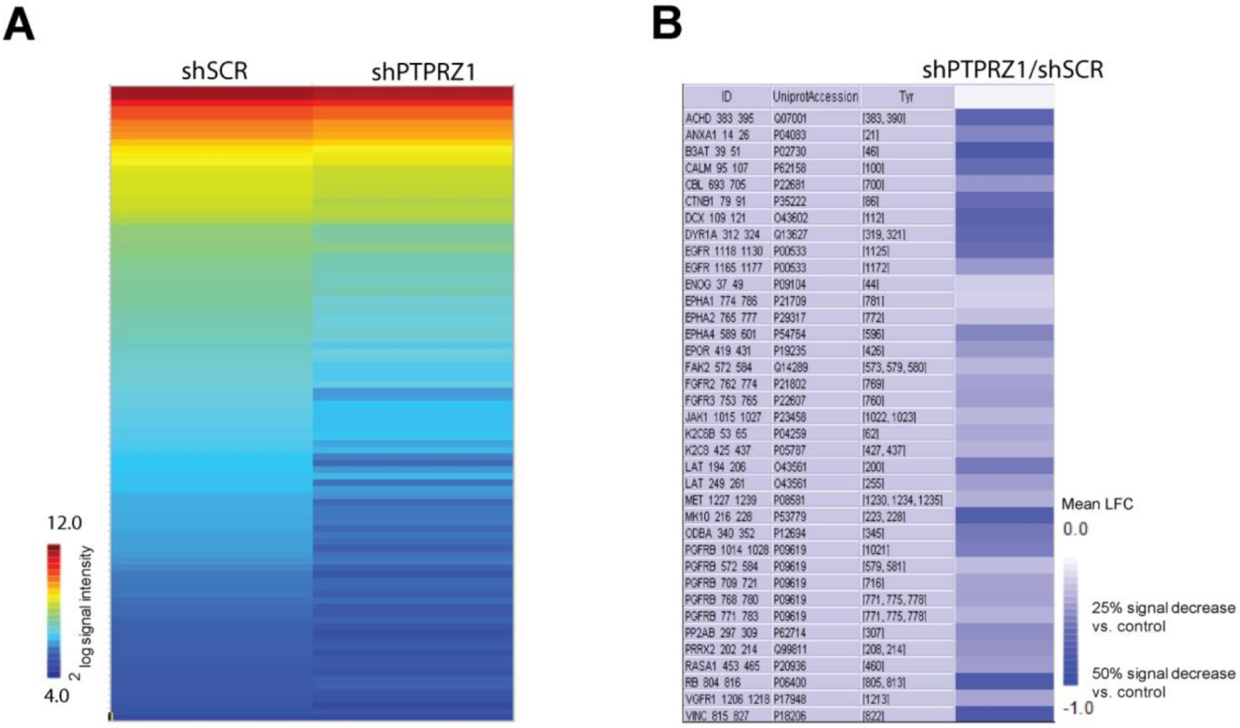

ID	UniprotAccession	Tyr
ACHD 383 395	Q07001	[383, 390]
ANXA1 14 26	P04083	[21]
B3AT 39 51	P02730	[46]
CALM 95 107	P62158	[100]
CBL 693 705	P22681	[700]
CTNB1 79 91	P35222	[86]
DCX 109 121	O43602	[112]
DYR1A 312 324	Q13627	[319, 321]
EGFR 1118 1130	P00533	[1125]
EGFR 1165 1177	P00533	[1172]
ENOG 37 49	P09104	[44]
EPHA1 774 786	P21709	[781]
EPHA2 765 777	P29317	[772]
EPHA4 589 601	P54764	[596]
EPOR 419 431	P19235	[426]
FAK2 572 584	Q14289	[573, 579, 580]
FGFR2 762 774	P21802	[769]
FGFR3 753 765	P22607	[760]
JAK1 1015 1027	P23458	[1022, 1023]
K2C6B 53 65	P04259	[62]
K2C8 425 437	P05787	[427, 437]
LAT 194 206	O43561	[200]
LAT 249 261	O43561	[255]
MET 1227 1239	P08581	[1230, 1234, 1235]
MK10 216 228	P53779	[223, 228]
ODBA 340 352	P12694	[345]
PGFRB 1014 1028	P09619	[1021]
PGFRB 572 584	P09619	[579, 581]
PGFRB 709 721	P09619	[716]
PGFRB 768 780	P09619	[771, 775, 778]
PGFRB 771 783	P09619	[771, 775, 778]
PP2AB 297 309	P62714	[307]
PRRX2 202 214	Q99811	[208, 214]
RASA1 453 465	P20936	[460]
RB 804 816	P06400	[805, 813]
VGFR1 1206 1218	P17948	[1213]
VINC 815 827	P18206	[822]

Supplementary Figure S2: Kinase activity profiling of E98 cells stably expressing shSCR/GFP and shPTPRZ1/TagRFP constructs. A) Heatmap representation of the mean signals (n=4) for 144 different peptides on the Tyrosine Kinase PamChip® Array upon incubation with lysates from shSCR or shPTPRZ1-expressing E98 cells. The order of peptide signal depiction (top, highest signal in red; bottom, lowest signal in blue) was determined by that in the shSCR sample. B) List of 37 peptides that were significantly differentially phosphorylated by shSCR- and shPTPRZ1-expressing E98 lysates. The ID column contains the protein name and first and last amino acid position of the peptide. Corresponding UniProt accession numbers are shown in the second column, and column 'Tyr' lists the positions of the phosphorylatable tyrosines in the peptide. The final column is a color-coded representation of the lower signals obtained with shPTPRZ1 E98 extracts as compared to the scrambled control.

Supplementary Table S1: Oligonucleotide sequences used in generating plasmid constructs.

Oligonucleotide (set)		Sequence
**1: CMV-TagRFP**	Forward	5'- GGCTCGAGTAGTTATTAATAGTAATC -3'
	Reverse	5'- CCTCTAGATCAATTAAGTTTGTGCCCC -3'
**2: CMV-EFGP**	Forward	5'- GGCGCTCTAGATAGTTATTAATAGTAATCAATTACG -3'
	Reverse	5'- GGCGCTCTAGATTACTTGTACAGCTCGTCCATG -3'
**3: PGK promoter**	Forward	5'- GGCATCGATCTCGAGCTACCGGGTAGGGGAGGCGC -3'
	Reverse	5'- GCCGCATCGATGTCGAAAGGCCCGGAGATG -3'
**4: shPTPRZ1**	Forward	5'- CACCGGAAGTGAATTCTCTGGAAAGCGAACTTTCCAGAGAATTCACTTCC -3'
	Reverse	5'- AAAAGGAAGTGAATTCTCTGGAAAGTTCGCTTTCCAGAGAATTCACTTCC -3'
**5: shSCR**	Forward	5'- CACCGTCAAACTGCTTACTCTAGACGAATCTAGATGTAAGCAGTTTGAC -3'
	Reverse	5'- AAAAGTCAAACTGCTTACATCTAGATTCGTCTAGATGTAAGCAGTTGAC -3'
**6: NotI-XhoI adapter**	Forward	5'- TCGACGCGGCCGCGAATTCCCGGGATCCACCGGTCTCGAGAAGCTTATCG ATACCGT -3'
	Reverse	5'- CTAGACGGTATCGATAAGCTTCTCGAGACCGGTGGATCCCGGGAATTCGC GGCCGCG -3'
**7: *Sst*I linker**		5'- AGCTGACCGCGGTC -3'
**8: PTPRZ-B cDNA**	Forward	5'- GCGCGCGGCCGCCCACTCTGAGAAGCAGAGGAG -3'
	Reverse	5'- GCGCCCGCGGGTGTTAAACTAAAGACTCTAAG -3'
**9: mutagenesis PTPRZ-B rescue**	Forward	5'- CAAGAGGAAGTGAATTTTCTGGAAAGGGTGATG -3'
	Reverse	5'- CATCACCCTTTCCAGAAAATTCACTTCCTCTTG -3'
**10: mutagenesis PTPRZ-B C/S**	Forward	5'- CCTGTTGTCGTCCACTCGAGTGCTGGAGTTGG -3'
	Reverse	5'- CCAACTCCAGCACTCGAGTGGACGACAACAGG -3'
**11: mutagenesis *Kpn*I full-length PTPRZ-B**	Forward	5'- GCTGAGAGCTTAGAGTCTTTGGTACCCGCGGTCAGCTTATCGAT -3'
	Reverse	5'- ATCGATAAGCTGACCGCGGGTACCAAAGACTCTAAGCTCTCAGC -3'
**12: *Kpn*I VSV-stop**	Forward	5'- CTTATACAGACATAGAGATGAACCGACTTGGAAAGTAGGTAC -3'
	Reverse	5'- CTACTTTCCAAGTCGGTTCATCTCTATGTCTGTATAAGGTAC -3'
**13: mutagenesis *Kpn*I PTPRZ-B ectodomain**	Forward	5'- GTTGGAATCCGAGAAGAAGGCGGTACCCCTTGTGATCGTGTCAGC -3'
	Reverse	5'- GCTGACACGATCACAAGGGGTACCGCCTTCTTCTCGGATTCCAAC -3'

1. Claes A, Schuuring J, Boots-Sprenger S, Hendriks-Cornelissen S, Dekkers M, van der Kogel AJ, Leenders WP, Wesseling P and Jeuken JW. Phenotypic and genotypic characterization of orthotopic human glioma models and its relevance for the study of anti-glioma therapy. Brain Pathol. 2008; 18(3):423-433.
2. Lee J, Kotliarova S, Kotliarov Y, Li A, Su Q, Donin NM, Pastorino S, Purow BW, Christopher N, Zhang W, Park JK and Fine HA. Tumor stem cells derived from glioblastomas cultured in bFGF and EGF more closely mirror the phenotype and genotype of primary tumors than do serum-cultured cell lines. Cancer Cell. 2006; 9(5):391-403.
3. Caretti V, Zondervan I, Meijer DH, Idema S, Vos W, Hamans B, Bugiani M, Hulleman E, Wesseling P, Vandertop WP, Noske DP, Kaspers G, Molthoff CF and Wurdinger T. Monitoring of tumor growth and post-irradiation recurrence in a diffuse intrinsic pontine glioma mouse model. Brain Pathol. 2011; 21(4):441-451.
4. Kreis TE. Microinjected antibodies against the cytoplasmic domain of vesicular stomatitis virus glycoprotein block its transport to the cell surface. The EMBO journal. 1986; 5(5):931-941.
5. Lamprianou S, Chatzopoulou E, Thomas JL, Bouyain S and Harroch S. A complex between contactin-1 and the protein tyrosine phosphatase PTPRZ controls the development of oligodendrocyte precursor cells. Proc Natl Acad Sci U S A. 2011; 108(42):17498-17503.
6. Cuppen E, Wijers M, Schepens J, Fransen J, Wieringa B and Hendriks W. A FERM domain governs apical confinement of PTP-BL in epithelial cells. Journal of cell science. 1999; 112 (Pt 19):3299-3308.
7. Schmidt F, van den Eijnden M, Pescini Gobert R, Saborio GP, Carboni S, Alliod C, Pouly S, Staugaitis SM, Dutta R, Trapp B and Hooft van Huijsduijnen R. Identification of VHY/Dusp15 as a regulator of oligodendrocyte differentiation through a systematic genomics approach. PLoS One. 2012; 7(7):e40457.
8. Livak KJ and Schmittgen TD. Analysis of relative gene expression data using real-time quantitative PCR and the 2(-Delta Delta C(T)) Method. Methods (San Diego, Calif. 2001; 25(4):402-408.
9. Noordman YE, Augustus ED, Schepens JT, Chirivi RG, Rios P, Pulido R and Hendriks WJ. Multimerisation of receptor-type protein tyrosine phosphatases PTPBR7 and PTP-SL attenuates enzymatic activity. Biochim Biophys Acta. 2008; 1783(2):275-286.
10. Navis AC, Hamans BC, Claes A, Heerschap A, Jeuken JW, Wesseling P and Leenders WP. Effects of targeting the VEGF and PDGF pathways in diffuse orthotopic glioma models. J Pathol. 2011; 223(5):626-634.
11. Sikkema AH, Diks SH, den Dunnen WF, ter Elst A, Scherpen FJ, Hoving EW, Ruijtenbeek R, Boender PJ, de Wijn R, Kamps WA, Peppelenbosch MP and de Bont ES. Kinome profiling in pediatric brain tumors as a new approach for target discovery. Cancer Res. 2009; 69(14):5987-5995.
